# Cultural Adaptation of the Modified Version of the Conflicts Tactics Scale (M-CTS) in Mexican Adolescents

**DOI:** 10.3389/fpsyg.2019.00619

**Published:** 2019-03-21

**Authors:** Rosa Carolina Ronzón-Tirado, Marina Julia Muñoz-Rivas, María Dolores Zamarrón Cassinello, Natalia Redondo Rodríguez

**Affiliations:** Department of Biological and Health Psychology, Universidad Autónoma de Madrid, Madrid, Spain

**Keywords:** dating violence, psychological testing, validity, cultural adaptation, Mexican adolescents

## Abstract

Several scales are used in Dating Violence studies assuming cross-cultural invariance and equivalence of the measures without making the proper validation in the intended populations. This study focuses on the importance of adapting existing dating violence psychological instruments (as the widely recognized Modified Version of the Conflict Tactics Scale, M-CTS) in diverse adolescent populations adjusting to international validation procedures that ensure the cultural fit of the instrument and the measurement invariance of the construct. We sought to adapt the M-CTS in Mexican adolescents (*N* = 1861; 57.5% woman) following the ITC Guidelines for Translating and Adapting Test. We made an analysis of the linguistic and cultural variables, followed by a Confirmatory Factor Analysis, and the evaluation of Construct and Known Groups Validities. We culturally modified six items and verified the four-factorial structure of the questionnaire proposed in previous studies (argumentation, psychological aggression, mild physical aggression, and sever physical aggression). We also found significant correlations in between the scores of the M-CTS and the Aggression Questionnaire (AQ) and the Dominating and Jealous Tactics Scale (DJTS), verifying the Construct Validity of the M-CTS to measure aggressive behaviors. Conclusion: the cultural adaptation of the M-CTS offered adequate reliability and validity scores in Mexican population expanding the possibilities of comparing prevalences of the problem between nations with a reliable instrument based on the same theoretical and methodological perspectives.

## Introduction

Psychometric test are not always adapted properly before they are used within two different cultures (Gjersing et al., 2010; Borsa et al., 2012). Researchers usually change test instructions, response formats, or the number and content of the items without taking into account if the modifications are suitable for the new context or consistent with the original version. Although these are probably well-intention actions based on the strong psychometric properties of the original instruments, they end up compromising the quality of the results (Eremenco et al., 2005; Reichenheim and Moraes, 2007).

Aware of this lack of rigor in the use of measurement tools, organizations such as the American Educational Research Association, the American Psychological Association, the European Federation of Psychologist Association, and the International Test Commission have generated guidelines in the last two decades for the development, administration, validation, and psychometric tests adaptation. Specifically, since 1976, the ITC has focused its efforts on the validation process (Oakland et al., 2009; Muñiz et al., 2015) and has edited a specific journal on the subject since 1998 (Hambleton and Patsula, 1999). It has also published the *ITC Guidelines for Translating and Adapting Tests* in International Test Commission [ITC] (2005), and its version 2.4 (2016), which main object has been to stablish a reliable method to cross-culturally adapt, administrate, and interpret tests.

Despite these important advances in the adaptation field, the most widely used scales still those specifically developed for the English-speaking population (Byrne and Van de Vijver, 2010; Muñiz et al., 2013). Testing the scales’ psychometric properties in other cultures or countries is necessary for the progress of research in topics that had been widely recognized as public health concerns (World Health Organization [WHO], 2002) such as teen dating violence.

During the last 5 years, there has been an increase in descriptive dating violence studies in Latino American cultures (Rodríguez, 2014; Celis-Sauce and Rojas-Solís, 2015; Boira et al., 2017; Rey-Anacona et al., 2017; Rojas-Solís et al., 2017). These studies, however, have not focused on using instruments adapted to the intended populations, making comparisons between groups difficult and hindering more concluding results. Specifically in Mexico, a remarkable variability has been found in the prevalence of documented aggressions during dating relationships, ranging from 46 to 86% of cases (Peña-Cárdenas et al., 2013; Carrillo-Flores, 2014; Vega-Valero, 2015; Oliva-Zárate et al., 2018). The available data is not conclusive and differs in terms of the theoretical models and methodologies used, as well as in the selection of the measurement instruments, which are generally created ex professor for each case and which psychometric properties are not usually reported.

In addition, it should be noted that the documented prevalence of teen dating violence in Mexico, as in other countries, has mainly been carried out in a global manner, without analyzing the directionality of the different behavioral expressions of the aggressions (Rubio-Garay et al., 2012). Few studies have discriminated the experiences of victimization/perpetration or have differentiated between verbal aggressions, mild physical aggressions and severe physical aggressions. Therefore, validate internationally recognized measurement instruments of dating violence, is an important contribution to recognize the magnitude of the problem and its characteristics, as well as for the development of prevention programs and intervention of violence in relationships in the Latin American context (Fernández-Fuentes et al., 2011; Fernández-González et al., 2013; Rubio-Garay et al., 2017).

Among the most widely used instruments for measuring teen dating violence in Latino America, the modified version (Cascardi et al., 1999) of the M-CTS (Neidig, 1986), stands out as one of the most appropriate scales to respond to the current demand for cross-cultural and multilingual evaluation of the problem (Ryan, 2013). This, unlike other scales, has shown adequate psychometric properties in previous adaptations in the United States (Straus, 2004), Italy (Nocentini et al., 2011), and Spain (Muñoz-Rivas et al., 2007a).

Although, the M-CTS has already been validated in Spanish-speaking population (Muñoz-Rivas et al., 2007a), there is still a lack of adaptations for Latin American countries. It would be a mistake to assume the permanence of the psychometric guarantees of the Spain validation in the rest of the Spanish-speaking countries. Applying the M-CTS without taking into account cultural variables between nations, could imply that the data obtained do not really reflect the reality of the adolescents, but the discrepancy in the understanding of the teen dating violence mediated by cultural and temporal variables such as religion, lifestyle and values. As well as, discrepancies originated by physical characteristics of the M-CTS like the item format and material of the test (Gjersing et al., 2010; International Test Commission [ITC], 2016).

For example, Latinos are said to hold more traditional attitudes about women, relationships and commitment, and Mexicans may have more rigid expectations about gender roles than North American or European populations. Although this kind of believes are changing and may vary across urban and rural groups, the powerful subjective influence of these believes over dating violence measure most be recognized (Hokoda et al., 2006; Shaffer et al., 2018).

In addition, when performing cross-cultural comparative studies, the variants found may not show the similarities or differences between countries, but the deficiencies of the M-CTS when evaluating each population mediated by the use of the language, such as, family structure of the language or semantic equivalence (Eremenco et al., 2005). Ryan et al. (1999) for example, found a lack of measurement equivalence when they attempted to apply attitudes surveys in a multinational organization where Spanish and Mexican employees worked. To reduce the lack of invariance they needed to make two Spanish versions of the surveys. After the adjustments, the wording of the items of each version clearly differed although the items represented similar content.

The objective of this study was to adapt the M-CTS Spanish version (Muñoz-Rivas et al., 2007a) in Mexican adolescents following internationally accepted guidelines proposed by International Test Commission [ITC] (2016). We hypothesize (a) to confirm the reliability and validity of the adapted M-CTS to measure different types of aggression in Mexican teen dating relationships. (b) that the cultural adaptation of the M-CTS would maintain the four-factor structure proposed in previous validations; (c) that the cultural adaptation of the M-CTS could discriminate different scores based on sex and age of the respondents; and that (d) that the M-CTS would correlate significantly with other scales that measure general aggression such as Aggression Questionnaire (AQ; Buss and Perry, 1992) and psychological violence in adolescents such as the Dominating and Jealous Tactics Scale (DJTS, Kasian and Painter, 1992).

## Materials and Methods

### Participants

The sample comprised 1,861 adolescents from six public schools in Xalapa (Veracruz, México). Inclusion criteria were (a) having had or currently having a dating relationship, (b) being between 12 and 18 years old (c) fluent Spanish reading and understanding (d) not presenting developmental disabilities incompatible with the requirements of the survey administration. 57.5% were women and 42.5% men, with a mean age of 15.5 years (*SD* = 1.39, range = 12–18), 47.6% of them were early adolescents (ages 12–15) and 52.4% late adolescents (ages 16–18). While 38% of the participants reported having a dating relationship with an average duration of 9.25 months (*SD* = 10.4), 62% reported not dating anyone currently but having done before (*M* = 5.82 months, *SD* = 7). The 91% reported having a heterosexual orientation, 7.1% bisexual, and 1.9% homosexual. Data was collected by convenience sampling method during the 2017–2018 school period.

### Instruments

Participants completed a questionnaire composed of sociodemographic and dating relationships data, as well as the instruments listed below:

*The Modified Conflict Tactics Scale* (M-CTS; Neidig, 1986) Spanish adaptation (Muñoz-Rivas et al., 2007a), is made up of 18 bidirectional items with a 5-point response format, ranging from 1 (*never*) to 5 (*very often*), assesses perpetration and victimization of psychological and physical violence. The answer frame of the question refers to the current relationship or last one in the case that the respondent do not have a relationship by the survey moment. It has a four-factor structure (i.e., argumentation; psychological violence; mild physical violence; and severe physical violence); and, in the Spanish adaptation, reliability, measured through Cronbach’s alpha coefficient in the subscales of Aggression, ranged from 0.65 to 0.82 for Perpetration and from 0.63 to 0.82 for Victimization (Muñoz-Rivas et al., 2007a). Scores interpretation: all the items have the same direction, each punctuation of the 8 subscales, indicates whether the respondent has been involved in such conduct, such as the frequency of the aggression in the reference period. The individual items can be examined together with the total scores of the subscales by the different implications that they could have, as an example, give a slap in comparison with punching.

The *Dominating and Jealous Tactics Scale* (DJTS; Kasian and Painter, 1992), Spanish validation (Muñoz-Rivas et al., 2019) has been used to analyze the convergent validity of M-CTS in measuring perpetration and victimization of psychological violence in courtship. It is made up of 11 bidirectional items with a 5-point response (from 1 “never” to 5 “very frequently”) to measure perpetration and victimization of dominant and jealous tactics. In the Spanish adaptation the reliability of the scale was good for both perpetration and victimization (Cronbach α = 0.76 and α = 0.78, respectively; Muñoz-Rivas et al., 2019). In the present sample, the result of the Exploratory Factor Analysis indicated that the eleven items, for both perpetration and for victimization scales were distributed in two factors (Dominant and Jealous tactics), the total variance explained by the two factors in the perpetration model was 38.1%, and 41.85% for the victimization model. The reliability of the perpetration scale was α = 0.77 and α = 0.82 for victimization scale, whit *α-*values for the domination and jealous scales between 0.67 and 0.79.

The *Aggression Questionnaire* (AQ; Buss and Perry, 1992), Spanish version (Andreu et al., 2002) is comprised of 29 Likert-type items with five response options (from 1 “totally agree” to 5 “totally disagree”) grouped into four factors: physical aggression (α = 0.86), verbal aggression (α = 0.86), anger (α = 0.86), and hostility (α = 0.86). It has been used in order to evaluate the convergent validity of the M-CTS to measure levels of general aggressiveness. In the present sample, the AQ scale obtained an Exploratory Analysis of the AQ Scale indicated, as in the Spanish validation, that the 29 items were distributed in 4 factors (physical aggression, verbal aggression, anger and hostility). The total variance explained by the 4 factors were 38,61%. The reliability of the verbal aggression scale was α = 0.68, α = 0.76 for physical aggression scale, α = 0.72 for anger scale, and α = 0.77 hostility.

Although DJTS and AQ have not been adapted yet to Mexican adolescents, they have been used to test convergent validity of the M-CTS in this study due to: (a) the lack of adapted Mexican scales to measure this constructs (López-Cepero et al., 2015) and, (b) their proven strong psychometric properties in English-speaking and Spanish young adults and adolescents samples (Cascardi et al., 1999; Muñoz-Rivas et al., 2007b, 2009; Chaín-Pinzón et al., 2012; Cascardi and Avery-Leaf, 2015).

### Procedure

The methodology proposed in the *ITC Guidelines for Translating and Adapting Test* (International Test Commission [ITC], 2016) was followed to carry out the adaptation. Guidelines and procedural objectives are reflected in Table 1.

**Table 1:** Summary of the ITC guidelines for translating and adapting test (2016).

**Precondition guidelines**
PC-1 (1) Obtain the permission from the intellectual holder of the original scale.
PC-2 (2) Evaluate that the amount of overlap in the definition and content of the construct measured by the test and the item content in the populations of interest is sufficient for the intended use.
PC-3 (3) Minimize the influence of any irrelevant cultural and linguistic differences (e.g., religion).
**Test development guidelines**
TD-1 (4) Ensure that the translation and adaptation process consider linguistic, psychological, and cultural differences in the intended populations (ask experts on the subject).
TD-2 (5) Use appropriate translation designs and procedures to maximize the suitability of the test adaptation. Focus on functional rather than on a literal equivalence.
TD-3 (6) Provide evidence that the test instructions and item content have similar meaning for the intended populations.
TD-4 (7) Provide evidence that the item formats, rating scales, scoring categories, test conventions, modes of administration, and other procedures are suitable for the intended populations.
TD-5 (8) Collect pilot data on the adapted test to enable item analysis, reliability assessment, and small-scale validity studies. Make any necessary changes.
**Confirmation guidelines**
C-1 (9) Select sample with characteristics and sufficient size for the intended use and relevance for the empirical analyses.
C-2 (10) Provide relevant statistical evidence about the construct equivalence, method equivalence, and item equivalence.
C-3 (11) Provide evidence supporting the norms, reliability, and validity of the adapted version.
C-4 (12) Use an appropriate equating design and data analysis procedures when linking score scales from different language versions.
**Administration guidelines**
A-1 (13) Minimize any culture- and language-related problems that are caused by administration procedures and response modes.
A-2 (14) Specify testing conditions that should be followed closely in all interest populations.
**Score scales and interpretation guidelines**
SSI-1 (15) Interpret any group score differences with reference to all relevant available information.
SSI-2 (16) Only compare scores across populations when the level of invariance has been established on the scale on which scores are reported.
**Documentation guidelines**
Doc-1 (17) Provide technical documentation of any changes.
Doc-2 (18) Provide documentation for test users that will support good practice in the use of the adapted test in the context of the new population.


The questionnaire was administered during school hours with prior informed consent of the participants, their parents, and the school’s supervisors and principals. Before the administration, the researchers provided participants information about the aims of the research, procedures, confidentiality protections, and participants’ right to withdraw the study. The classrooms were designated as sample units, and the approximate response time of the questionnaire participants was 50 min. The evaluators were trained in the use of the scale by both the authors of the Spanish version and Mexican researchers.

Descriptive statistics and departure from the normality of the variables were made follow by Exploratory Factor Analyses (EFA) using General Least Square (GLS) method of estimation and reliability test for AQ and DJTS scales (both scales have been used to test the convergent validity of the M-CTS). Afterwards, *Mann–Whitney U test* were performed to asses difference between M-CTS scores by sex and age, effect size was measured with *A static*. Then Spearman correlations were made between subscales to test convergent validity of the M-CTS. All of these analyses were made using the statistical package, SSPS v20 (IBM, 2011).

Finally, the Structural Equation Models were tested using the Mplus 7.0 software (Muthén and Muthén, 1998–2015) Due to the distribution of the variables MLM estimator was used. To study model-fit, the following indexes and values were considered (Jöreskog, 2001; Hooper et al., 2008): Root Mean Square Error of Approximation (Good fit = 0 ≥ RMSEA ≤ 0.05; Acceptable fit = 0.05 ≥ RMSEA ≤ 0.08); Standardized Root Mean Square Residual (Good fit = 0 ≥ RMSEA ≤ 0.05; Acceptable fit = 0.05 ≥ RSMR ≤ 0.1) and Comparative Fit Index (Acceptable Fit = CFI ≥ 0.9). Reliability of the M-CTS Subscales was measured using *Cronbach’s Alpha* and *Omega* coefficients.

## Results

The results obtained for each phase indicated in the ITC Guidelines are described in this section (International Test Commission [ITC], 2016; Table 1).

### Precondition Guidelines

The license to use the scale was obtained from the authors of the Spanish version of the M-CTS (Muñoz-Rivas et al., 2007a), and researchers obtained the approval of the Research Ethics Committee of the Autonomous University of Madrid to carry out the study (CEI-85-1576). Subsequently, two dating violence experts (i.e., Spanish and Mexican postdoctoral researchers with more than 10 years of experience on the topic and several published studies about dating violence) qualitatively analyzed the instrument to verify the equivalence of the construct and to minimize the influence of cultural variables (e.g., lifestyles and value systems) in both populations. The evaluation was positive, and no modifications were necessary.

### Test Development Guidelines

Two independent postdoctoral Mexican researchers, experts in dating violence and skilled in psychometrics, made adaptations to the content of the scale. They focused on grammar, terminology, and the colloquial use of words to ensure that the adaptation process considered the cultural, psychological, and linguistic differences of Mexican adolescents (Borsa et al., 2012). They agreed on the modification of items 6, 8, and 14, (in perpetration and victimization scales). In item 6, “estabais” was replaced by “estaban”; in item 8, “picar” and “picarte” were replaced by “molestar” and “molestarte”; and in item 14, “abofeteado” by “dar una cachetada.” Once the scale was modified, the authors of the Spanish version verified that the proposed modifications did not alter the construct.

To empirically support the modifications, a pilot test of the scale was conducted using a sample of 118 adolescents randomly selected from two educational centers in Xalapa. The sample was made up of 50.8% women and 42.2% men with ages between 12 and 17 years (*M* = 14.81 years; *SD* = 1.42). The reliability of the scale was analyzed using the Cronbach’s Alpha coefficient and Confidence Intervals 95%, in all cases the coefficient provided statistically acceptable scores similar to those obtained in the Spanish version (i.e., α = 0.46 CI [0.26–0.61] and 0.44 CI [0.24–0.60] for argumentation; 0.68 CI [0.58–0.77] and 0.59 CI [0.45–0.69] for verbal aggression; α = 0.81 CI [0.76–0.86] and 0.75 CI [0.68–0.82] for mild physical aggression; and, 0.76 CI [0.68–0.83] and 0.56 CI [0.40–0.68] for severe physical aggression, perpetration and victimization subscales).

In addition, the convergent validity of the test was analyzed using the AQ and DJTS scales. Positive and significant Spearman correlations were found for: (a) The M-CTS psychological violence subscales and DJTS dominant tactics subscales (*r*_s_ = 0.44, *p* < 0.001, for perpetration; and *r*_s_ = 0.45, *p* < 0.001, for victimization); (b) The M-CTS Psychological Violence subscales and the DJTS Jealous Tactics subscales (*r*_s_ = 0.49, *p* < 0.001, for perpetration; and *r*_s_ = 0.48, *p* < 0.001 for victimization); (c) The MCTS Psychological Violence subscales and the AQ Verbal Aggression subscale (*r*_s_ = 0.20, *p* < 0.001).

Positive significant Spearman correlations were also found between the subscales of the (a) M-CTS Mild Physical Violence perpetration subscale and the subscale of physical aggression of the AQ (*r*_s_ = 0.17, *p* < 0.001). There was no significant correlation in-between M-CTS Severe Physical Violence subscale and the AQ Physical Aggression subscale (*r*_s_ = 0.04, *p* = 0.054), this last result is explained by the items content of both subscales, since the level f aggressiveness is much higher u the items used in the M-CTS.

### Confirmation Guidelines

Once the pilot had concluded, the M-CTS was administered to a large sample of 1,861 adolescents from Xalapa. Results follow.

#### Reliability

The reliability of perpetration and victimization M-CTS subscales was estimated through the Cronbach’s Alpha coefficient and the Confidence Intervals 95% (CI 95%) for each case. The CI 95% was estimated to assess the precision of the α measures and determine between what values the α coefficient could oscillate in the population (Domínguez-Lara and Merino-Soto, 2015). The analysis revealed Cronbach’s Alpha scores between α = 0.43 for Argumentation on the victimization scale and α = 0.78 for Mild Physical Violence victimization. The coefficients values of Argumentation and Sever Physical aggression subscales were under 0.5 but still acceptable taking into account the scare number of items of each subscale (Crutzen and Ygram, 2017). Additionally, Omega coefficients were also calculated because it has been shown (Ventura-León and Caycho-Rodríguez, 2017) that unlike the coefficient of alpha, Omega provides more precise reliability measures as it works with factorial loads (Table 2).

**Table 2:** Cronbach’s alpha and omega coefficients of the M-CTS subscales.

	Perpetration			Victimization		
		
	α	CI 95%	ω	α	CI 95%	ω
Argumentation	0.45	0.4–0.49	0.48	0.43	0.38–0.47	0.43
Psychological violence	0.65	0.62–0.67	0.64	0.66	0.64–0.69	0.67
Mild physical violence	0.77	0.75–0.78	0.80	0.78	0.77–0.80	0.81
Severe physical violence	0.71	0.69–0.73	0.73	0.43	0.39–0.47	0.44


*α, Cronbach’s Alpha coefficient; ω, omega coefficient.*

Furthermore, given the importance of this instrument for professional and epidemiological practice, reliability between relevant groups have been calculated. Analysis in early adolescents subgroup reveled acceptable Cronbach’s Alpha scores between α = 0.78 [CI 0.32–0.48] for mild physical victimization and α = 0.58 [CI 0.50–0.65] for severe physical victimization, and values of 0.40 [CI 0.32–0.48] and 0.46 [CI 0.35–0.54] for perpetration and victimization argumentation subscale. Analysis in late adolescents reveled acceptable Cronbach’s Alpha scores between α = 0.65 [CI 0.63–0.68] for verbal aggression perpetration and α = 0.79 [CI 0.77–0.80] for mild physical victimization, and values of 0.46 [CI 0.41–0.51] and 0.42 [CI 0.37–0.47] for perpetration and victimization argumentation subscale. The results for argumentation subscales still acceptable considering the scare number of the items in each one.

#### Confirmatory Factor Analysis

Due to the distributions of the variables, the confirmatory factor analysis was conducted using the MLM maximum likelihood parameter with standard errors and a mean-adjusted chi-square test statistic that are robust to non-normality. Compared to de ML estimation, a robust MLM approach is less dependent of the assumption of multivariated normal distribution and have the advantage of computing robust versions of CFI and RMESEA. Thus, the use of MLM estimator was the most appropriate approach for the analysis (Byrne, 2012). The structural equation models were configured according to the four factor structure (for both perpetration and victimization scales) that previous studies had supported in North American (Caulfield and Riggs, 1992; Pan et al., 1994; Straus, 2004) and Spanish samples (Muñoz-Rivas et al., 2007a). Additionally two factor structure proposed by Cascardi et al. (1999) was tested, it was discarded do to its unacceptable fit indexes scores (CFI = 0.75, RMSEA = 0.038, and SRMR = 0.074 for perpetration; CFI = 0.91, RMSEA = 0.023, and SRMR = 0.051, for victimization).

Given the correlations within-factor errors and similar content in the items (Hooper et al., 2008), some modifications were made through the correlation of error terms to the four-factor model results (CFI = 0.84, RMSEA = 0.030, and SRMR = 0.047 for perpetration; CFI = 0.88, RMSEA = 0.027, and SRMR = 0.05, for victimization). The error term correlations included for the perpetration model were: item 6 with 7, from the psychological aggression factor; and error term 12 with 14; and 15 with 13, from the mild physical violence. For the victimization model: correlation between error terms 12 and 14, and 13 with 9 from the mild physical aggression factor.

The criteria to include this correlations in the model was the strength of the modification indices (MI) and Expected Parameter Change (EPC) values for the residual covariance, as well as the obvious overlap of the item contents (Byrne, 2012). For example, correlation between error terms 12 and 14, was include in both models (perpetration and victimization) due to it had MI values of 28.97 and 23.92, respectively; and the evident similarity of items content: 12 “You have hit your boyfriend/girlfriend” and item 14 “You have slapped your boyfriend/girlfriend.” Goodness-of-fit results of before (Model 1) and after the correlation of error terms (Model 2) that confirm the fit of the proposed models to the original version are presented in Table 3.

**Table 3:** Goodness-of-fit indexes used to assess confirmatory factor analysis for the M-CTS.

Model 1		
Index	Perpetration	Victimization
CFI	0.84	0.88
Number of free parameters	60	60
Root Mean Square Error Approximation (RMSEA)		
Estimate	0.030	0.027
Standardized Root Mean Square Residual (SRMR)		
Value	0.047	0.05

**Model 2 (Including correlation of error terms)**		
**Index**	**Perpetration**	**Victimization**

CFI	0.90	0.91
Number of free parameters	63	62
Root Mean Square Error Approximation (RMSEA)		
Estimate	0.024	0.024
Standardized Root Mean Square Residual (SRMR)		
Value	0.043	0.049


The final models obtained Good fit values in RMSEA and RSMR, and acceptable-fit values for CFI. It should be mention that the lack of convergence in the indexes values most not be understood as the model is misspecified or had any flaws in the data. It has been documented (Lai and Green, 2016) that this disagree arises because: (a) the two indexes by design, evaluate fit from different perspectives and, (b) the cut values of both are arbitrary and independent from each other.

Tables 4, 5 show the distribution of the items in each of the factors in perpetration and victimization models.

**Table 4:** Standardize model results: STDYX Standardization of the M-CTS.

	Squared
	multiple	Factor
Item	correlations	loading	Estimate/*SE*
Argumentation			
(1) ¿Tú has discutido de forma tranquila?	0.23	0.47	12.93***
(2) ¿Tú has buscado información para apoyar tu punto de vista?	0.42	0.65	16.20***
(3) ¿Tú has llamado o intentado llamar a otra persona para que te ayude a arreglar las cosas?	0.11	0.33	11.23***
Psychological violence			
(4) ¿Tú has insultado o maldecido a tu novio?	0.32	0.56	20.07***
(5) ¿Tú te has molestado al hablar de un tema y/o te has negado a hacerlo?	0.28	0.53	20.67***
(6) ¿Tú te has marchado molesto/a de la habitación de la casa o el lugar donde estaban discutiendo?	0.24	0.48	18.49***
(7) ¿Tú has llorado como consecuencia de una discusión?	0.19	0.44	15.43***
(8) ¿Tú has dicho o hecho algo para fastidiar o molestar a tu novio?	0.30	0.55	21.66***
Mild physical violence			
(9) ¿Tú has amenazado con golpear o lanzar algún objeto a tu novio/a?	0.31	0.55	11.99***
(10) ¿Tú has intentado sujetar físicamente a tu novio/a?	0.15	0.38	10.74***
(11) ¿Tú has lanzado algún objeto a tu no novio/a?	0.41	0.64	15.82***
(12) ¿Tú has golpeado a tu novio/a?	0.41	0.64	13.25***
(13) ¿Tú has empujado o agarrado a tu novio/a?	0.52	0.72	20.09***
(14) ¿Tú le has dado una cachetada a tu novio/a?	0.34	0.68	11.08***
(15) ¿Tú has pateado o mordido a tu novio/a?	0.31	0.56	14.68***
Severe physical violence			
(16) ¿Tú has intentado ahogar a tu novio/a?	0.23	0.48	2.26*
(17) ¿Tú has dado una paliza a tu novio/a?	0.55	0.74	8.52***
(18) ¿Tú has amenazado a tu novio con un cuchillo o algún arma?	0.67	0.82	5.17***


*Perpetration subscale.*

*^∗∗∗^Two-tailed p-value < 0.001; ^∗^Two-tailed p-value < 0.05.*

**Table 5:** Standardize model results: STDYX standardization of the M-CTS.

	Squared
	multiple	Factor
Item	correlations	loading	Estimate/*SE*
Argumentation			
(1) ¿Tu novio/a ha discutido de forma tranquila?	0.12	0.34	9.48***
(2) ¿Tu novio/a ha buscado información para apoyar su punto de vista?	0.27	0.52	14.75***
(3) ¿Tu novio/a ha llamado o intentado llamar a otra persona para que ayude a arreglar las cosas?	0.22	0.47	14.32***
Psychological violence			
(4) ¿Tu novio/a te ha insultado o maldecido?	0.30	0.55	18.91***
(5) ¿Tu novio/a se ha molestado al hablar de un tema y/o se ha negado a hacerlo?	0.28	0.53	21.93***
(6) ¿Tu novio/a se ha marchado/molesto/o de la habitación de la casa o el lugar donde estaban discutiendo?	0.33	0.57	24.56***
(7) ¿Tu novio/a ha llorado como consecuencia de una discusión?	0.21	0.46	18.34***
(8) ¿Tu novio/a ha dicho o hecho algo para fastidiarte o molestarte?	0.33	0.58	25.02***
Mild physical violence			
(9) ¿Tu novio te ha amenazado con golpearte o lanzarte algún objeto?	0.43	0.66	16.52***
(10) ¿Tú novio ha intentado sujetarte físicamente?	0.24	0.49	14.64***
(11) ¿Tú novio te ha lanzado algún objeto?	0.33	0.57	11.62***
(12) ¿Tú novio te ha golpeado?	0.38	0.62	14.78***
(13) ¿Tu novio te ha empujado o agarrado?	0.61	0.78	27.80***
(14) ¿Tu novio te ha dado una cachetada?	0.29	0.53	9.9***
(15) ¿Tu novio te ha pateado o mordido?	0.40	0.64	17.74***
Severe physical violence			
(16) ¿Tu novio te ha intentado ahogar?	0.25	0.49	3.9***
(17) ¿Tu novio te ha dado una paliza?	0.23	0.48	3.98***
(18) ¿Tu novio te ha amenazado con un cuchillo o algún arma?	0.16	0.40	3.56***


*Victimization subscale.*

*^∗∗∗^Two-tailed p-value < 0.001.*

#### Known Groups Validity

Due to the distribution of the variables *Mann–Whitney U test* were performed in order to assess the ability of the M-CTS to contrasts of hypotheses of equality between means by sex and age. Along with the estimation of the statistical differences, the effect size was calculated though *A* static with Hanley y McNeil method, values around 0.010, 0.30, and 0.50, were considered as small, medium, and large, respectively. Table 6 shows, as in previous studies (Fernández-Fuertes and Fuertes, 2010), significant statistical differences in scores between men and women. Higher levels of aggressiveness were self-reported by women in relation to men for the subscales of psychological violence (*Z* = 7.91; *p* < 0.001; *A* = 0.39) and mild physical violence (*Z* = 4.59; *p* < 0.001; *A* = 0.52). In the case of victimization, men self-reported significantly higher levels of victimization through psychological violence (*Z* = 2.17; *p* < 0.05; *A* = 0.52).

**Table 6:** Means, standard deviations (SD), statistical differences and effect size by sex in the M-CTS subscales.

	Women	Men	Total
	(*N* = 1070; 57.5%)	(*N* = 791; 42.5%)	(*N* = 1861)	*Z*	*A*
Perpetration	*M (SD)*	*M (SD)*	*M (SD)*		
Argumentation	5.03 (2.58)	4.90 (2.51)	5.12 (2.62)	1.90	
Psychological violence	5.25 (2.68)	5.11 (2.61)	4.56 (3.47)	7.91***	0.39
Mild physical violence	5.13 (3.66)	4.37 (3.24)	1.09 (2.30)	4.59***	0.44
Severe physical violence	3.79 (3.02)	4.81 (3.63)	0.02 (0.27)	1.65	
Victimization					
Argumentation	1.32 (2.59)	1.03 (2.20)	4.99 (2.56)	1.81	
Psychological violence	0.79 (1.78)	1.29 (2.75)	4.56 (3.42)	2.17*	0.52
Mild physical violence	0.03 (0.32)	0.03 (0.30)	1.14 (2.45)	1.64	
Severe physical violence	0.02 (0.18)	0.04 (0.30)	0.03 (0.30)	0.25	


*^∗^Two-tailed p-value p < 0.05; ^∗∗∗^Two-tailed p-value p < 0.001.*

*A values around 0.010, 0.30, and 0.50, were considered as small, medium, and large, respectively*.

To analyze the differences by age, the participants were grouped into early adolescence (12–14 years) and late adolescence (15–18 years) according to the criteria on the physical and mental development of the adolescents proposed by the United Nations International Children’s Emergency Fund (UNICEF, 2011). Consistent with previous studies’ findings (Foshee et al., 2009), the violent behaviors were self-reported more frequently by the group of late adolescents. Table 7 shows significant differences in the scales of perpetration in argumentation (*Z* = 2.92; *p* < 0.005; *A* = 0.55) and psychological violence (*Z* = 3.22; *p* < 0.001; *A* = 0.55).

**Table 7:** Means, SD and differences by age in the M-CTS subscales.

	12–14 years	15–18 years	Total
	(*n* = 370; 19.9%)	(*n* = 1491; 80.1%)	(*n* = 1861)	*Z*	*A*
Perpetration	*M (SD)*	*M (SD)*	*M (SD)*		
Argumentation	4.75 (2.59)	5.21 (2.62)	5.12 (2.62)	2.92**	0.55
Psychological violence	4.05 (3.33)	4.68 (3.49)	4.56 (3.47)	3.22***	0.55
Mild physical violence	1.10 (2.42)	1.09 (2.27)	1.09 (2.30)	0.52	
Severe physical violence	0.03 (0.28)	0.02 (0.27)	0.02 (0.27)	0.28	
Victimization	*M (SD)*	*M (SD)*	*M (SD)*		
Argumentation	4.73 (2.66)	5.05 (2.53)	4.99 (2.56)	2.16*	0.54
Psychological violence	5.05 (2.53)	4.70 (3.44)	4.56 (3.42)	3.85***	0.56
Mild physical violence	1.17 (2.55)	1.13 (2.43)	1.14 (2.45)	0.17	
Severe physical violence	0.02 (0.29)	0.04 (0.30)	0.03 (0.30)	0.91	


*^∗^Two-tailed p-value p < 0.05;^∗∗∗^Two-tailed p-value p < 0.001.*

*A values around 0.010, 0.30, and 0.50, were considered as small, medium, and large, respectively*.

Differences in the victimization self-reported aggressions are also shown in Table 7, there were significant differences for the subscales of argumentation (*Z* = 2.16; *p* < 0.05; *A* = 0.54) which had higher prevalences in late adolescents, and in the psychological violence which had higher prevalences in early adolescents (*Z* = 3.85; *p* < 0.001; *A* = 0.56).

#### Convergent Validity

Finally, Spearman correlations were calculated between M-CTS subscales, and for the scores of physical aggression and verbal aggression of the AQ scale with the perpetration subscales of the M-CTS, as well as the correlations between the DJTS subscales and the perpetration and victimization subscales of the M-CTS (Table 8). As expected, all correlations were statistically significant, except five; four of them from the perpetration subscales: (a) argumentation and severe physical violence, (b) argumentation and physical aggression of the AQ, (c) severe physical violence and verbal aggression subscale of AQ, and (d) severe physical violence and Jealous Tactics from DJTS. And, one from the victimization subscales (e) argumentation and severe physical violence from the M-CTS.

**Table 8:** Spearman correlations between the M-CTS and DJTS and AQ scales, Means, SD.

			Mild	Severe
		Psychological	physical	physical
	Argumentation	violence	violence	violence	*M*	*SD*
Perpetration						
Argumentation	–				5.12	2.62
Psychological violence	0.27***	–			4.56	3.47
Mild physical violence	0.07**	0.42***	–		1.09	2.30
Severe physical violence	-0.03	0.05*	0.17***	–	0.02	0.27
Dominating Tactics	0.13***	0.42***	0.31***	0.097***	1.10	2.04
Jealous Tactics	0.22***	0.47***	0.22***	0.04*	3.04	2.85
AQ-verbal aggression	0.056*	0.19***	0.16***	0.02	2.64	0.78
AQ-Physical aggression	-0.025	0.13***	0.16***	0.04*	2.35	0.78
Victimization						
Argumentation	–				4.99	2.56
Psychological violence	0.26***	–			4.56	3.42
Mild physical violence	0.09***	0.42***	–		1.14	2.45
Severe physical violence	-0.01	0.11***	0.21***	–	0.03	0.30
Dominating tactics	0.11***	0.45***	0.29***	0.12***	1.45	2.53
Jealous tactics	0.23***	0.48***	0.20***	0.06*	3.59	3.26


*Perpetration and Victimization.*

*^∗^Two-tailed *p*-value < 0.05; ^∗∗^Two-tailed p-value < 0.01; ^∗∗∗^Two-tailed p-value < 0.001.*

### Administration Guidelines

The following specifications are recommended to administrate the test. First, researchers should inform the participants about the objectives and purposes of the study. Second, the researchers must obtain the informed consent of the adolescents, parents or legal guardians, and school’s principals. Also, it is important that the researcher maintain the anonymity of participants’ responses to the test. The researcher should read the test instructions in groups and explain the answer format with an example (first item) and should resolve participants’ doubts before starting the test administration. Next, the results should be scored by two or three evaluators trained by experts per group. Finally, the researcher should allow 50 min for the test administration.

### Score Scales and Interpretation Guidelines

Once the reliability and validity of the M-CTS in Mexican adolescents were tested and found acceptable, the Mexican scale’s properties were qualitatively compared with those obtained by the Spanish version to identify the equivalence of the construct and factor structure consistence, in both populations. In both the Mexican and Spanish versions of the scale, the model of equations calculated through the confirmatory factor analysis obtained satisfactory scores in RMSEA, and CFI; this outcome verified the structural and functional statistics qualities of the scale in both populations.

## Discussion

The incorporation of the methodology proposed by the ITC to adapt the M-CTS for Mexican adolescents represents a remarkable advance for the dating violence research field in México. It makes possible—by contemplating cultural and linguistic variables of the nation—the consensual, rigorous, and reliable measurement of the problem. The results provide an indispensable base for the development of effective intervention and prevention programs (Borsa et al., 2012).

This adaptation represents, in addition, an improvement to the previous analysis of the M-CTS in the Spanish population; in the present study, in addition to a confirmatory factor analysis, known groups and concurrent validity analyses were conducted. These improvements provide greater evidence of the adequate psychometric guarantees and abilities of the M-CTS to respond to the current measurement demands of dating violence (Straus, 2004).

Nevertheless, it should be noted that as topic of future investigations, it would be interesting to test the measurement invariance of the M-CTS to ensure suitable group comparisons between men and women, or in between group ages. We strongly recommend to implement specific statistical procedures to test Differential Item Functions based on Classical Test Theory as Logistic Regressions or Lord Chi-square calculation based on the Item Response Theory, for example (Çokluk et al., 2016).

It is important to mention that the six modified items in this version proved to have adequate psychometric properties for measuring dating violence in Mexico because they obtained in each case a factorial weight above 0.40. The total scale and subscales obtained acceptable levels of reliability and validity and also demonstrated an equal factor structure to the one proposed in the literature and the previous validation studies (Fernández-González et al., 2013). These results position the M-CTS as one of the best scales for cross-cultural studies of dating violence.

After carrying out the adaptation, the usefulness of the methodology proposed by International Test Commission [ITC] (2016) was confirmed, as was the need for internationally recognized guides for the development and adaptation of scales. Otherwise, by continuing the use of the scales without carrying out the necessary adaptations—through proven and agreed procedures—for the populations of interest, there will be a great risk of reporting data that, instead of reflecting the problem, will report deficiencies in the scales, differences in the factorial structure, or measurement variances (Eremenco et al., 2005; Gjersing et al., 2010).

## Data Availability

The datasets generated for this study are available on request to the corresponding author.

## Ethics Statement

Ethical approval for all procedures involving human subjects and analyses conducted for the current manuscript was provided by the Research Ethics Committee of the Autonomous University of Madrid (CEI-85-1576) in accordance with federal regulations governing human subjects research and with the 1964 Helsinki declaration and its later amendments or comparable ethical standards. Informed consent was obtained from all individual participants, their parents, and school’s supervisors and principals.

## Author Contributions

All authors listed have made a substantial, direct and intellectual contribution to the work, and approved it for publication.

## Conflict of Interest Statement

The authors declare that the research was conducted in the absence of any commercial or financial relationships that could be construed as a potential conflict of interest.
